# Supplementary material for: A Multifunctional and Fast-Response Lysosome-Targetable Fluorescent Probe for Monitoring pH and Isoxaflutole
Source: Int J Mol Sci. 2022 Jun 2;23(11):6256. doi: 10.3390/ijms23116256 (PMC9181397; doi:10.3390/ijms23116256)
Supplement: Supplementary file 1 [file ijms-23-06256-s001.zip › ijms-1716663-supplementary.pdf]

Electronic Supplementary Information

A multifunctional and fast-response lysosome-targetable fluorescent probe for monitoring pH and isoxaflutole

Liu Yang [†], *Yan Liu* [†], *Mingli Yue* [†], *Ping Li* [†], *Yulong Liu* [†], *Fei Ye* [*,†], *and Ying Fu* [*,†]

[†]*Department of Applied Chemistry, College of Arts and Sciences, Northeast Agricultural University, Harbin 150030, P. R. China.*

**Corresponding Author: Fei Ye, Email address: yefei@neau.edu.cn*

**Corresponding Author: Ying Fu, Email address: fuying@neau.edu.cn*

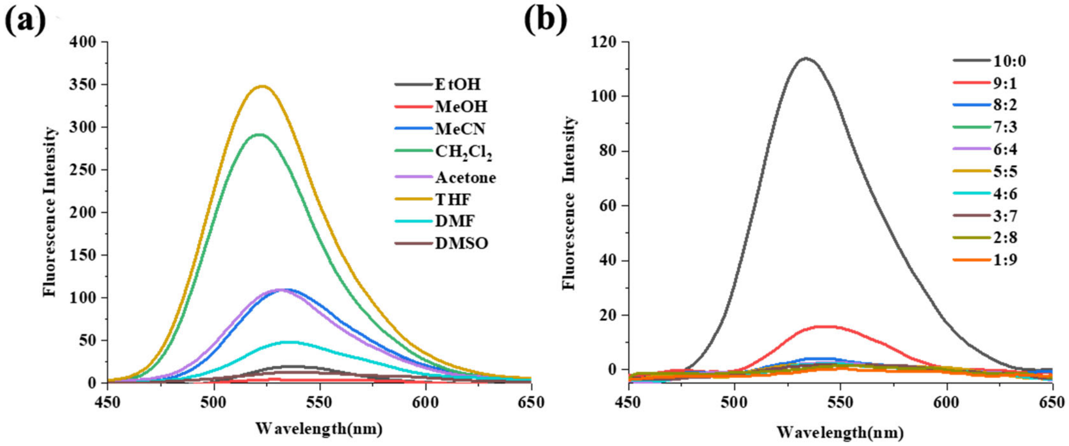

Figure S1. Solvent effect (a) and fluorescence intensity of different proportions of acetonitrile and water (b) of **MMN** at room temperature.

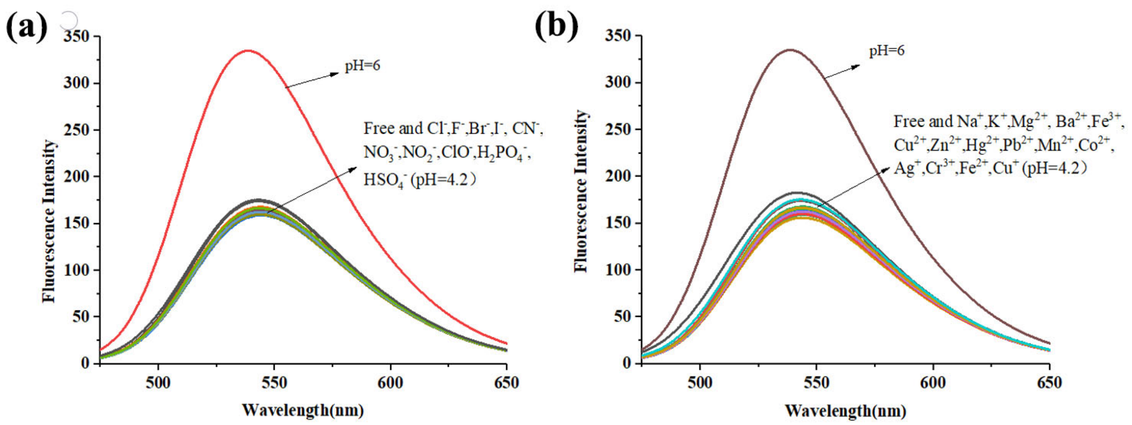

Figure S2. (a) The fluorescence spectra of **MMN** with or without various metal ions in CH_3CN/H_2O (*V*/*V*, 9/1) at pH 4.2 at room temperature.

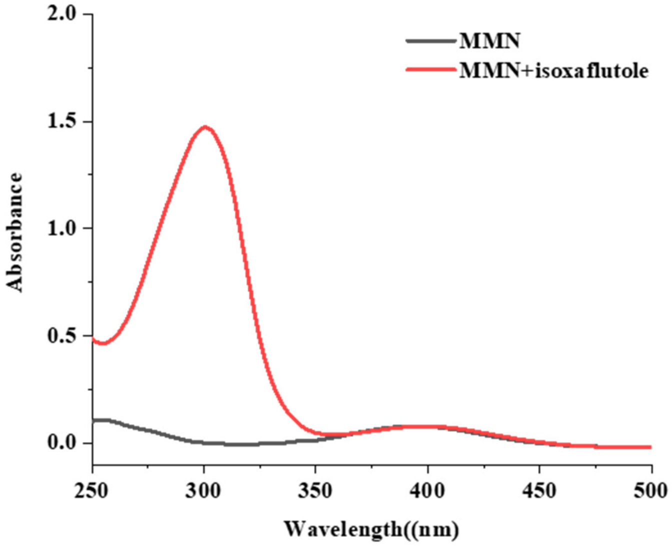

Figure S3. UV-Vis spectra change of **MMN** (10 μM) in CH_3CN solution with or without isoxaflutole (50 μM) at room temperature.

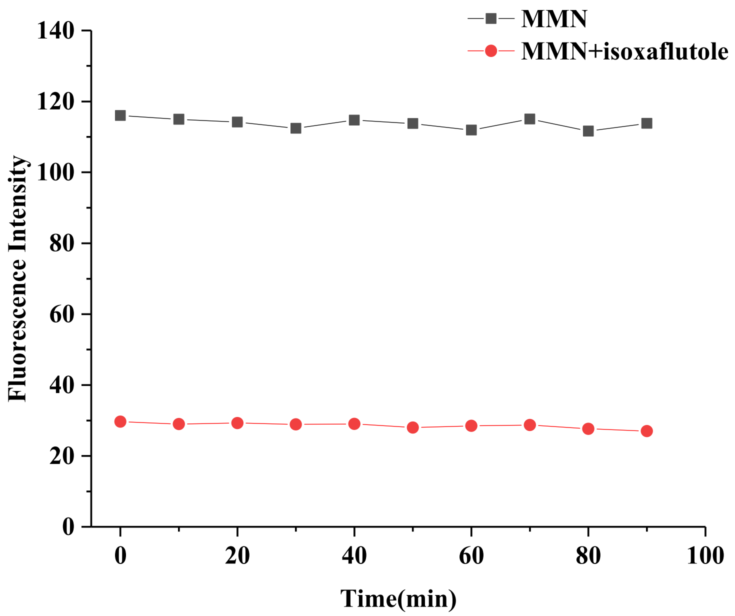

Figure S4. Fluorescence intensity of **MMN** in the absence and presence of isoxaflutole in CH_3CN solution.

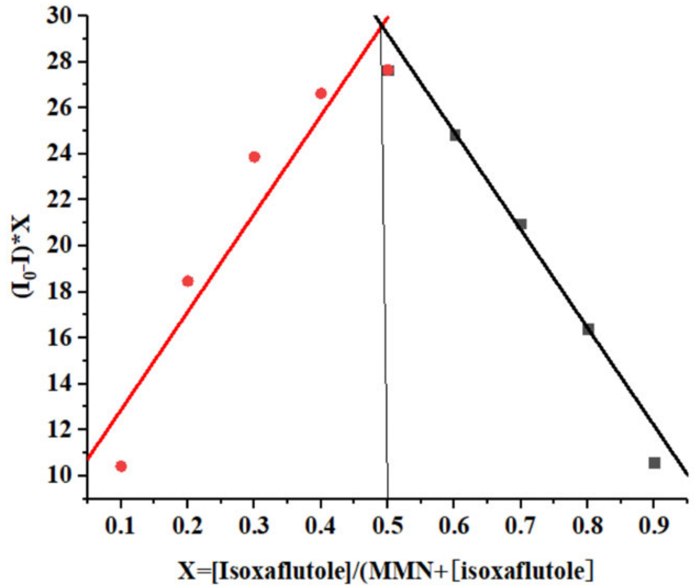

Figure S5. The Job's plot of **MMN** with isoxaflutole determined by fluorescence spectra in CH_3CN solution.

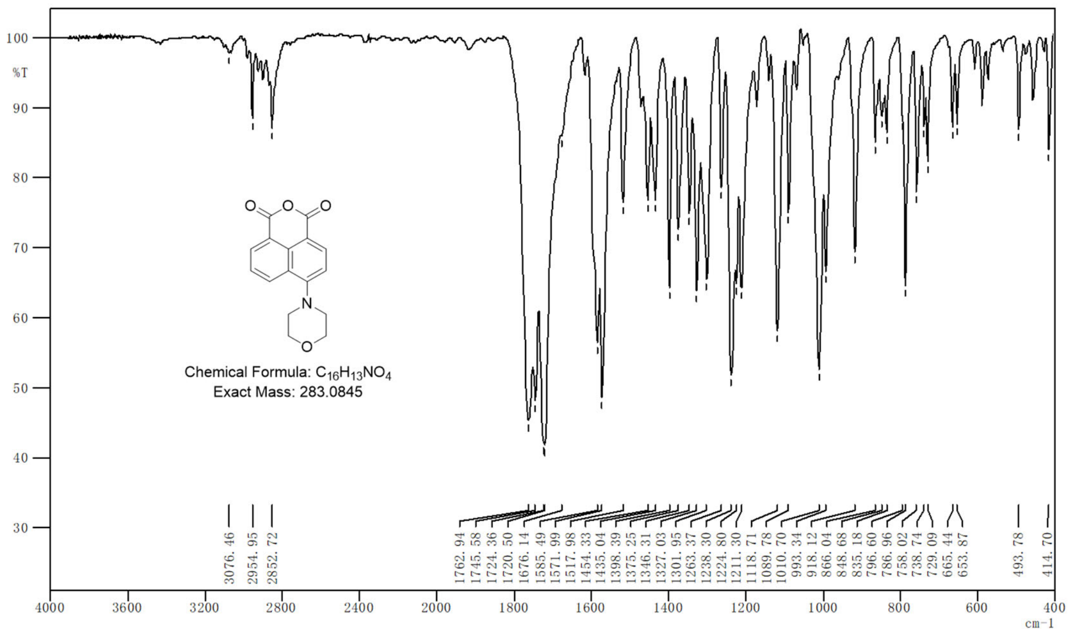

Figure S6. IR spectrum of compound **1**.

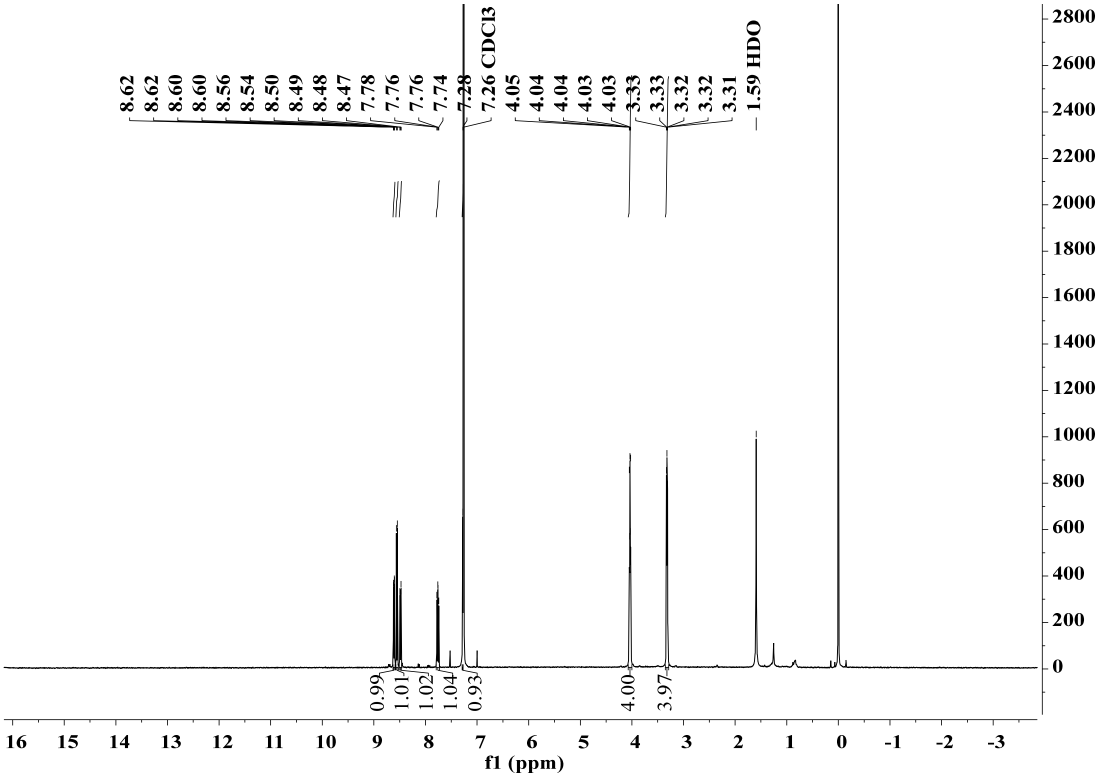

Figure S7. ^{1}H NMR spectrum of compound **1**.

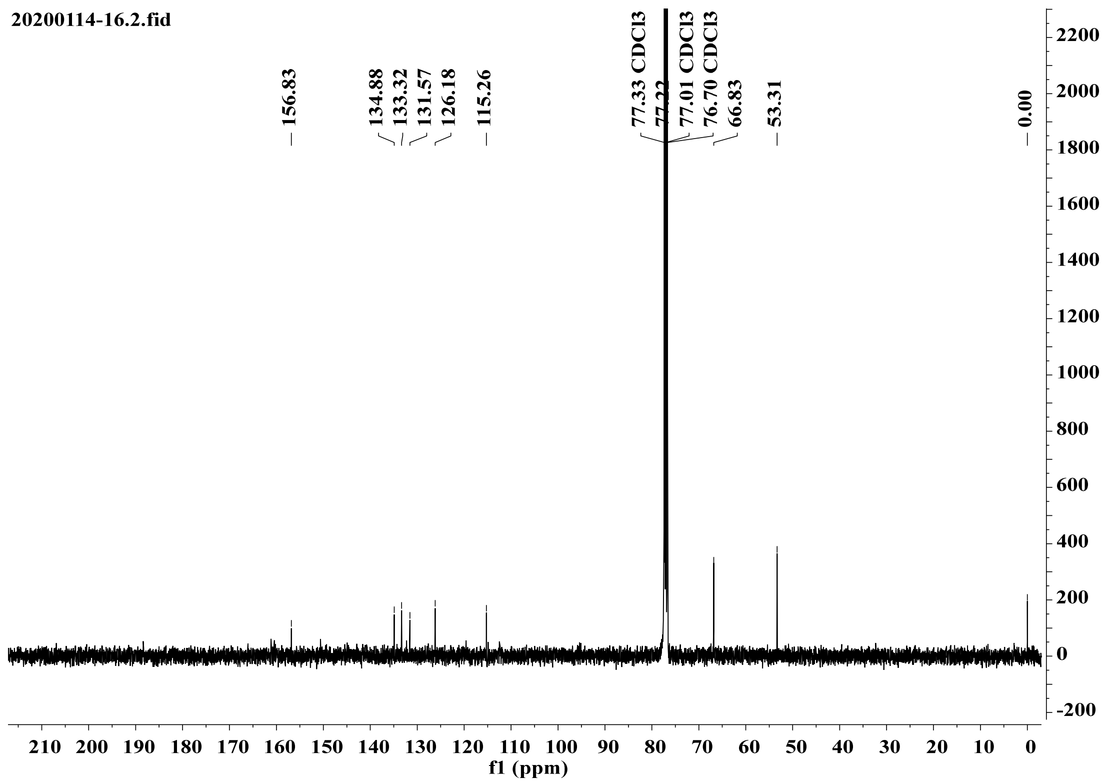

Figure S8. ^{13}C NMR spectrum of compound **1**.

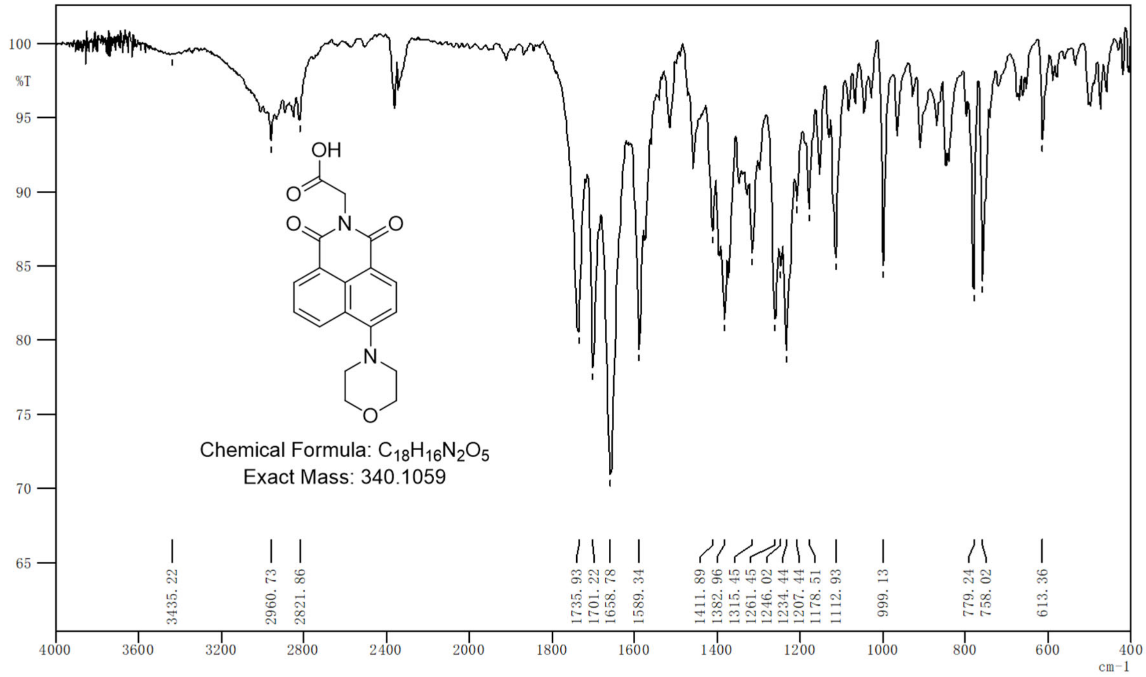

Figure S9. IR spectrum of compound **2**.

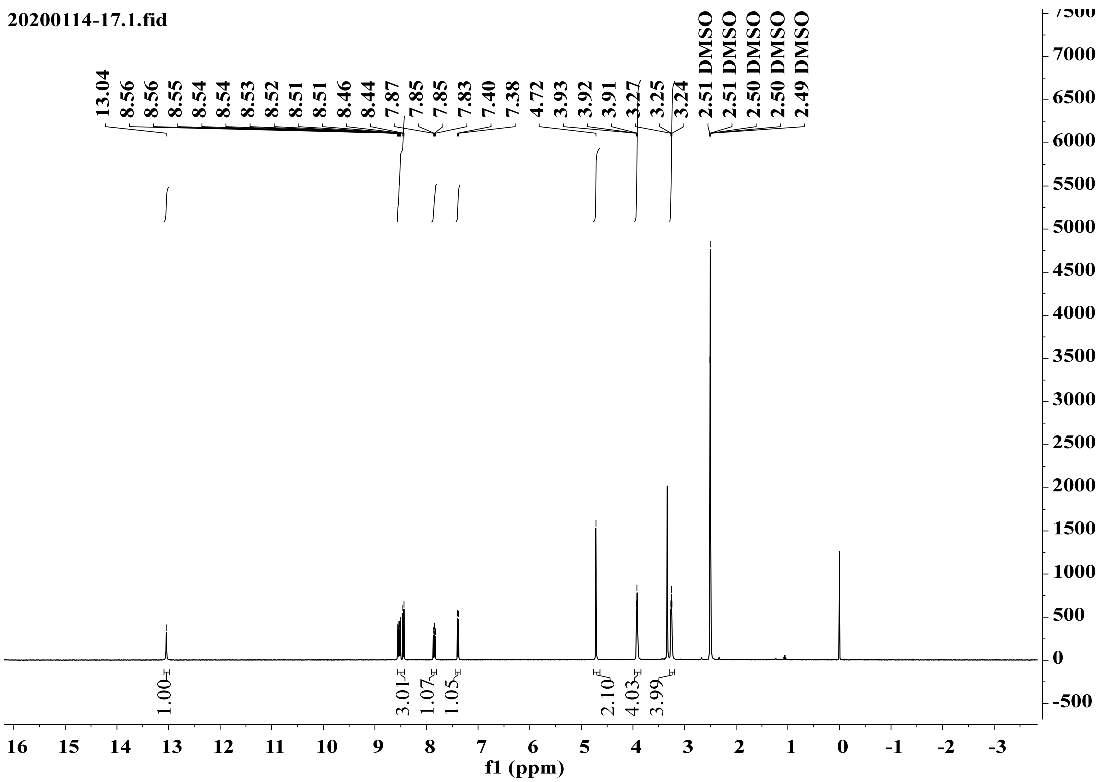

Figure S10. ^{1}H NMR spectrum of compound **2**.

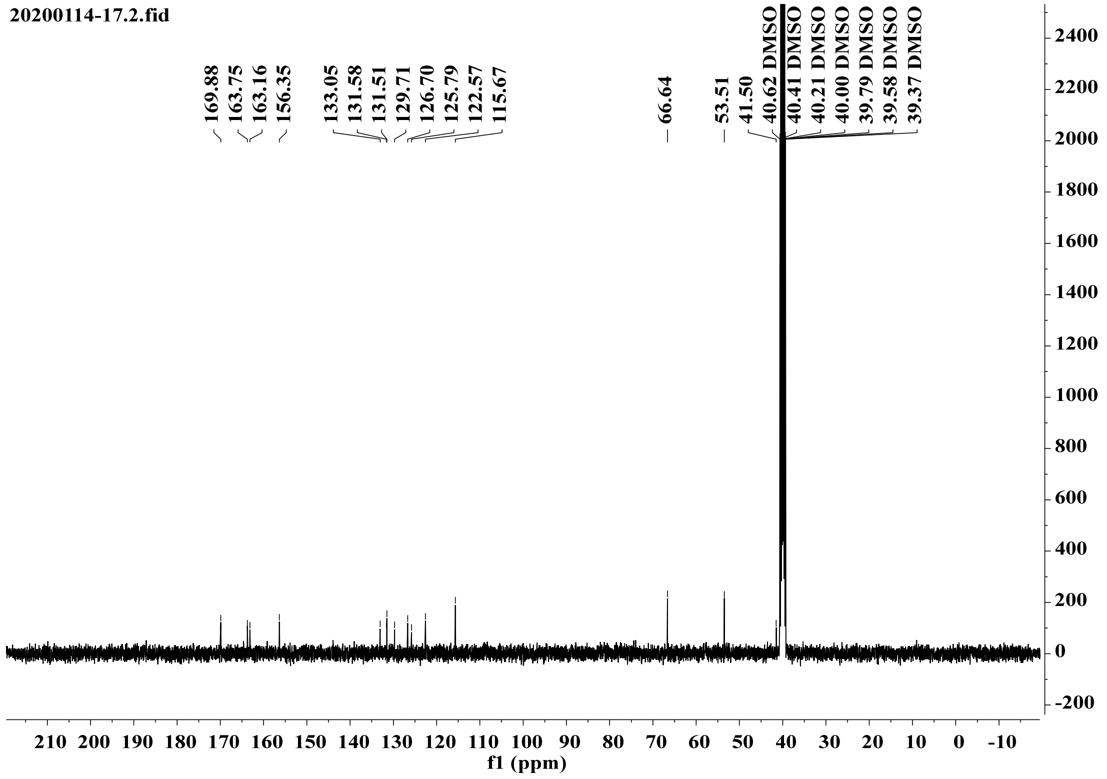

Figure S11. ^{13}C NMR spectrum of compound **2**.

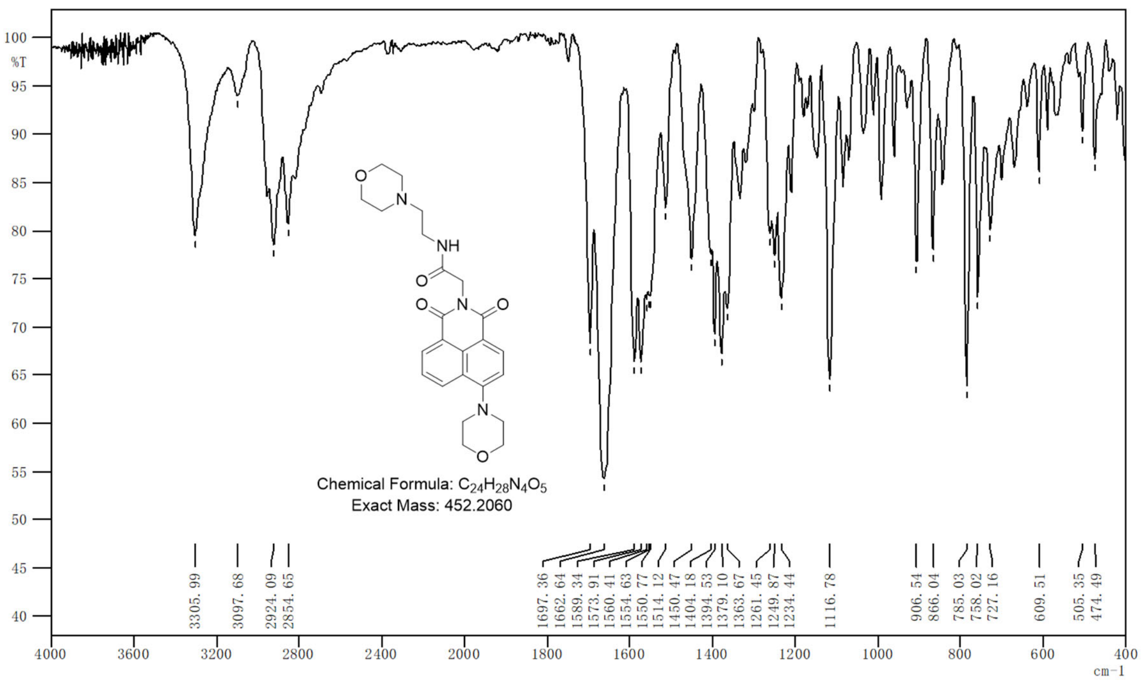

Figure S12. IR spectrum of compound **MMN**.

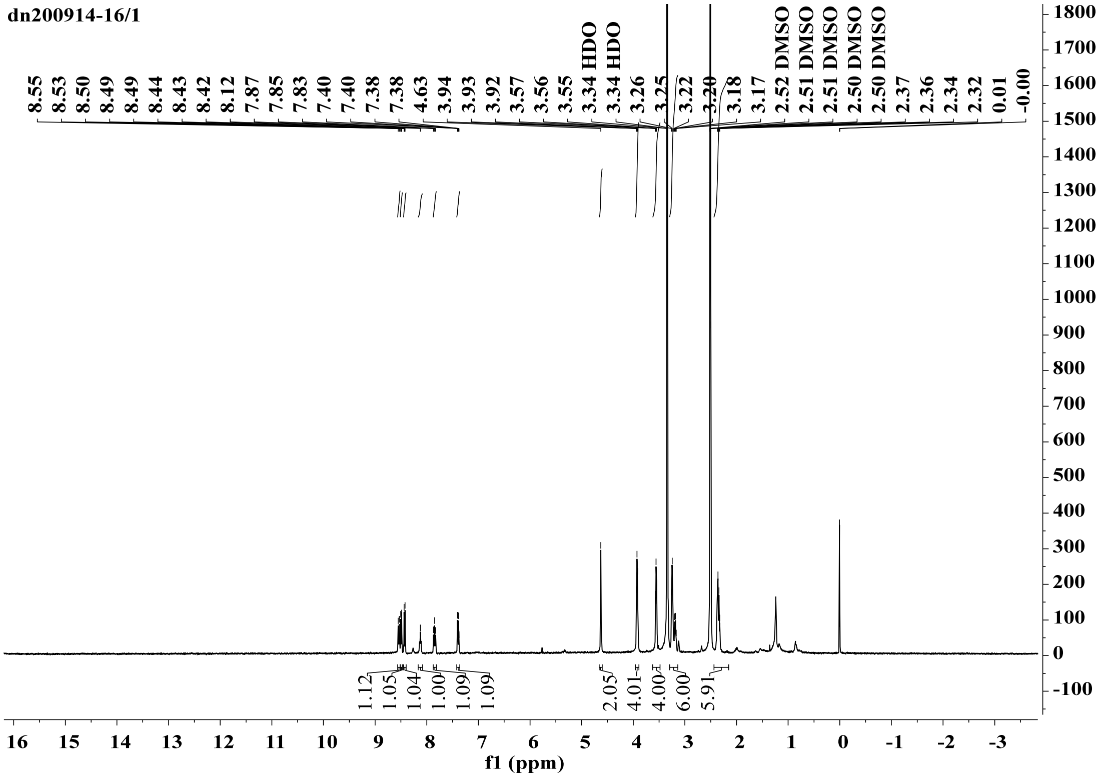

Figure S13. ^{1}H NMR spectrum of compound **MMN**.

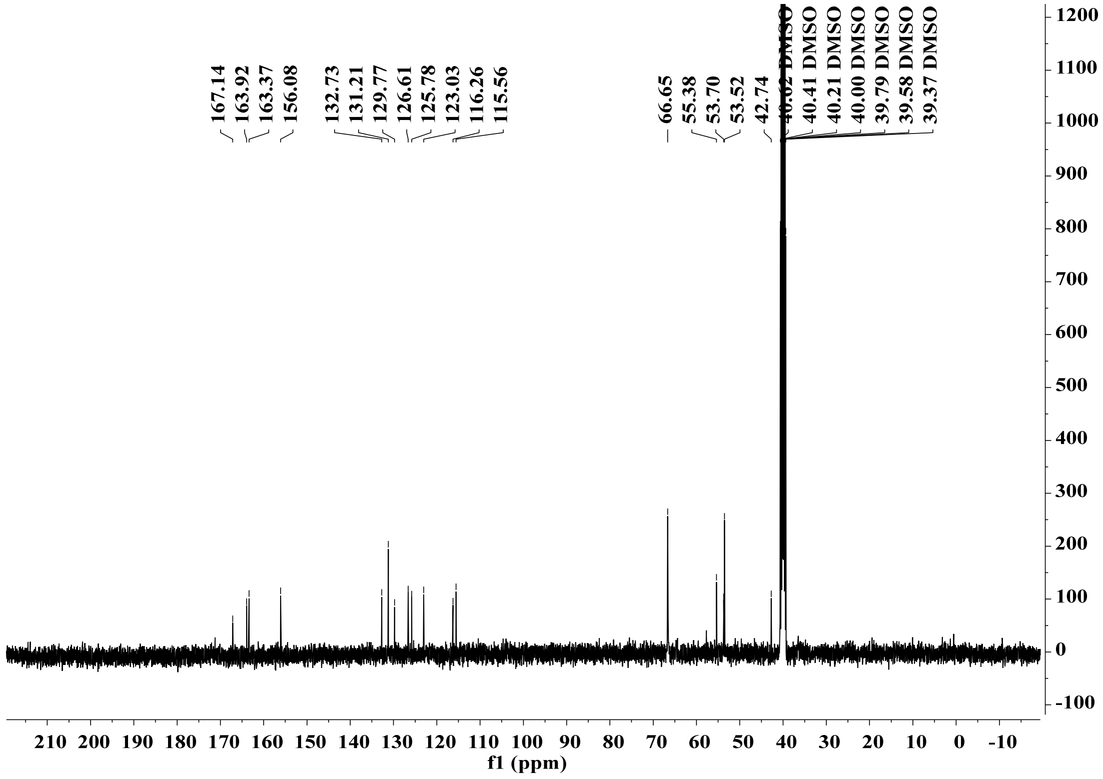

Figure S14. ^{13}C NMR spectrum of compound **MMN**.

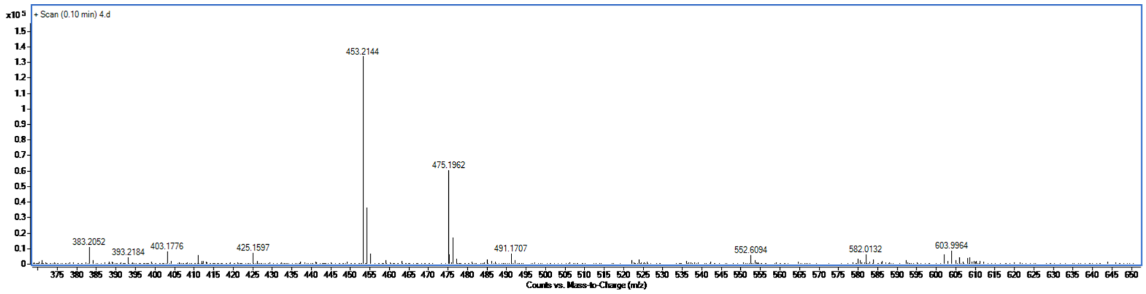

Figure S15. Mass Spectrum (M+H$^+$) of compound **MMN**.

Table S1. The comparison of binding constants with other reported fluorescent probes.

Type	Probe	Binding constant (K) (M^{-1})	Detecting pesticides	Ref.
Off-on	**MMN**	3.7×10^5	Isoxaflutole	This work
On-off	**Rho B@1**	9.08×10^4	nitenpyram	[1]
Off-on	**Tb@UiO-66**	5.36×10^2	teflubenzuron	[2]
On-off	**RhB@Zr-MOF**	9.01×10^4	nitenpyram	[3]

References:

1. L. Yang, Y.L. Liu, C.G. Liu, F. Ye, Y. Fu, Two luminescent dye@MOFs systems as dual-emitting platforms for efficient pesticides detection, J. Hazard. Mater. **2020**, 381, 120966.

2. L. Yang, Y.L. Liu, X.X. Ji, C.G. Liu, Y. Fu, F. Ye, A novel luminescent sensor based on Tb@UiO-66 for highly detecting Sm^{3+} and teflubenzuron, J. Taiwan Inst. Chem. Eng. **2021**, 126, 173–181.

3. L. Yang, Y.L. Liu, C.G. Liu, Y. Fu, F. Ye, A built-in self-calibrating luminescence sensor based on RhB@Zr-MOF for detection of cations, nitro explosives and pesticides, RSC Adv. 2020, 10, 19149–19156.
